# Does Money Matter? Characteristics Associated With Joint Pain Medication Access Among Older Adults

**DOI:** 10.1093/geroni/igab046.411

**Published:** 2021-12-17

**Authors:** Aviad Tur-Sinai, Netta Bentur, Jennifer Shuldiner

**Affiliations:** 1 Yezreel Valley College, Mevasseret Zion, Yerushalayim, Israel; 2 Tel Aviv University, Tel Aviv University, Tel Aviv, Israel; 3 University of Toronto, University of Toronto, Ontario, Canada

## Abstract

The experience of pain is a widespread phenomenon among adults, especially older adults, and entails high costs to both individuals and society. The objective of the current research is to determine if the ability to pay and supplementary insurance are factors associated with pain medication among individuals over 50. Data came from Survey of Health, Aging and Retirement in Europe (SHARE). The sample included 64,281 individuals 50+ from nineteen European countries and Israel. Joint pain was common with one out of three reporting joint pain. Prevalence of pain was similar among different age groups, and more women reported joint pain. Among those in pain, about 21.5% of the individuals reported mild pain, 52.9% moderate and 26% severe pain. In the multivariate logistic regression, we found that men and those older than 60 suffered more from joint pain, while controlling for education and subjective assessment of the ability to cope economically (Able to make ends meet). A large percentage of those with pain were not taking medication to manage their pain, and there were significant demographic differences between those that did and did not take medication. Those that took medication were younger, male, had more education, were able to cope economically and had supplementary insurance. Our study showed that about half of the individuals with pain were not taking medication to manage their pain. Our results demonstrate that among individuals over 50 in Europe income is strongly associated with taking pain medication and that there is economic inequity in medication access.

